# Perspectives on the Parathyroid–Thymus Interconnection—A Literature Review

**DOI:** 10.3390/ijms26136000

**Published:** 2025-06-23

**Authors:** Maria-Paula Comănescu, Otilia Boișteanu, Delia Hînganu, Marius Valeriu Hînganu, Roxana Grigorovici, Alexandru Grigorovici

**Affiliations:** 1 Department of Surgery I, Faculty of Medicine, Grigore T.Popa University of Medicine and Pharmacy, University Street No. 16, 700115 Iași, Romania; maria-paula.comanescu@umfiasi.ro (M.-P.C.); ale.grigorovici@umfiasi.ro (A.G.); 2 Discipline of Anesthesiology and Intensive Care, Department of Surgery, Faculty of Dental Medicine, Grigore T.Popa University of Medicine and Pharmacy, University Street No. 16, 700115 Iași, Romania; otilia.boisteanu@umfiasi.ro; 3 Department of Morpho-Functional Sciences I, Faculty of Medicine, Grigore T.Popa University of Medicine and Pharmacy, University Street No. 16, 700115 Iași, Romania; 4 Faculty of Medicine, Grigore T.Popa University of Medicine and Pharmacy, University Street No. 16, 700115 Iași, Romania; grigorovici.roxana@d.umfiasi.ro

**Keywords:** thymic function, parathyroid glands, parathyroid–thymus interconnection, morfofunctional endocrine research

## Abstract

The parathyroid and thymus glands are key components of the endocrine and immune systems, respectively, with intriguing developmental, anatomical, and functional interrelationships. This study starts from the hypothesis that, given their shared embryological origin, it is plausible that the thymus and parathyroid glands interact functionally and may share pathological pathways. The present study explores the developmental pathways, spatial proximity, and potential cross-talk between these glands. Recent studies suggest that parathyroid hormone (PTH) may influence thymic function, including T-cell maturation and immune regulation, while thymic signaling molecules could impact calcium homeostasis and parathyroid activity. Understanding the functional and etiopathogenical relations between these endocrine glands offers new insights into endocrine–immunological crosstalk, and therapeutic approaches targeting disorders such as hypoparathyroidism, thymomas, myasthenia gravis and thymic hypoplasia. Perspectives and conclusion: Future research is essential to discover the molecular mechanisms underpinning this dynamic interrelation and its broader implications for health and disease. Because there is still very little data on this interaction, in-depth studies are necessary on large groups of patients. This research proposes a cross-study of the receptors for the main substances secreted by the two categories of endocrine glands. At the same time, it is essential to carry out an in-depth study on the cervico-pericardial ligaments through the lens of this glandular interaction. These ligaments could contain the main blood and nerve communication pathway between the parathyroids and the glands.

## 1. Introduction

The threshold of our research consists of the common embryological development of these two endocrine glands—the thymus and inferior parathyroids [1,2]. The two categories of glands have their embryological origin in the pharyngeal pouches of the III and IV arches. Retrospective research on this subject led us to the idea that it is very likely that these glands are related both functionally and physio-pathologically [3,4,5].

However, the primary functions of the thymus and parathyroid glands are quite distinct, with the thymus playing a critical role in producing T cells and the parathyroid controlling calcium physiology through the production of the parathyroid hormone (PTH) [6].

A stable serum calcium level is important, among other things, for the proper functioning of smooth and skeletal muscles, membrane depolarizations through which neurotransmission is carried out, and the secretion of enzymes and hormones [7,8,9]. This is regulated by the parathyroid glands, endocrine organs located in the vicinity of the thyroid gland [10,11,12] at the cervical level. The parathyroid glands express receptors (CaSR) that monitor serum calcium levels (CaSR) and regulate their metabolism through negative feedback mechanisms. When serum calcium levels decrease, the parathyroids secrete PTH, which interacts with G protein-coupled receptors on bone cells to release calcium from long bones into the bloodstream [13,14].

Recent advances in understanding molecular mechanisms point to early thymic organogenesis, prior to the expression of the transcription factor *Foxn1*. This is an early marker of thymic epithelial cell identity and exhibits a series of independent developmental stages, including the specification of the pharyngeal endoderm, patterning of the pharyngeal pouches, and positioning towards thymic fate, with particular emphasis on epithelial–mesenchymal interactions [15,16].

This hormone also acts at the renal level, where PTH increases calcium reabsorption in the ascending limb of the loop of Henle and in the distal tubule, as well as the excretion of inorganic phosphate in the urine [17,18].

Regarding the physio–pathological correlation, there is evidence of this interrelationship. In terms of parathyroid involvement with the thymus in early organogenesis, prior to the separation of the two organs, the parathyroid domain expresses Ccl21, a chemokine that contributes to help recruit lymphoid cells to the thymus.

On the other hand, *Gcm2* (a parathyroid-specific transcription factor crucial for parathyroid gland development, known as GCMB or hGCMb, and a homolog of the Drosophila glial cells missing gene) is specifically required only in parathyroid organogenesis, but is still present after parathyroidectomies led to the assumption that thymus could act as a secondary source of PTH. This was proven to be inaccurate. Later research explained this phenomenon by proving that the thymus–parathyroid separation is “messy”, with clusters of parathyroid cells remaining throughout the neck region and transdifferentiating in small cervical thymi that produce the PTH. *Foxn1* and *Gcm2* transcription factors are cell context dependent and they each require permissive transcription factor landscapes in order to successfully interfere with organ-specific cell fate [19].

Research shows that thymic epithelial cells presenting only acetylcholine receptor (AChR) subunits “prime” T-helper cells, thereby prompting antibody development against thymic myoid cells (which express fully formed, clustered AChR). These, in turn, will diversify to recognise intact muscle AChR.

Recent studies found that the parathyroid hormone prompts human T-cell activation; PTH acts on T cells, and acute exposure to PTH augments PHA-induced T-cell proliferation and IL-2 production [20]. This action of PTH is related to its biological activity and is most likely due to the ability of PTH to enhance the entry of calcium into cells [21]. This means there may be a third correlation, as T cells are activated by PTH, which in turn activate AChR AB formation [22,23].

All of this evidence on the thymus–parathyroid interrelationship highlights three converging directions in molecular research: the common developmental origins of these glands, functional communication pathways, and etiopathogenic mechanisms. The aim of our study is to consolidate existing data on these three research domains and to outline future strategies for their investigation.

## 2. Material and Methods

The following international databases were used for this study: Web of Science, PubMed (National Library of Medicine and NIH), SciELO, SpringerLink, ScienceDirect, ResearchGate, Wiley Online Library, Lippincott Williams & Wilkins, MDPI and Bond University Research Portal. The selection of articles was carried out using a targeted search by keywords and specific inclusion/exclusion criteria. In these databases (excluding Web of Science), we searched the three study directions mentioned.

A comprehensive analysis was performed, which included categorical assessments, assessments of heterogeneity, investigations of publication bias and subgroup analyses, with an emphasis on biomolecular techniques. All selected articles were in English and open access.

Article types were limited to original research articles and reviews from the Web of Science categories of surgery, developmental studies, evolutionary biology, anatomy and morphology, and microscopy. Exclusion criteria omitted studies not related to thymic and parathyroid surgery, musculoskeletal disorders, cell biology, autoimmune diseases, molecular biology, genetics, and imaging technologies. Only publications from 2019 to 2025 were considered. Records were excluded based on predefined criteria, such as lack of relevance to the thymus–parathyroid axis, absence of experimental or clinical data, or insufficient methodological clarity.

For the Web of Science, the search was refined to studies focused on cellular and molecular biology, biomolecules, cell differentiation, immunology, and anatomical research. This three-pronged approach ensured a robust data set for comprehensive exploration of the topic (available in Supplementary Materials).

1. The inclusion criteria for the development of the glands were based on keywords such as molecular research on thymic organogenesis and molecular research on parathyroid gland organogenesis.
2. Regarding the functional relationship between these, we used the following keywords: thymus function, parathyroid and thymus function, T lymphocytes and Ca.
3. To highlight the etiopathogenic relationship, we used the following keywords: thymic cause of hypoparathyroidism, parathyroid and thymic dysfunctions, causes of myasthenia gravis.

Although this is a narrative review, the search strategy and selection process were conducted in accordance with PRISMA 2020 guidelines, adapted for non-systematic reviews [24].

## 3. Results

For the developmental research on thymic and parathyroid glands, we found 51 + 3 articles. Regarding the functional relationship, our research found the following: thymus function, 891 articles; parathyroid and thymus function, one article; and lymphocytes T and Ca, 1666 articles. For the etiopathogenic relationship, we found 5 + 2 + 485 articles (Table 1).

### 3.1. Common Development Molecular Research on Thymic and Parathyroid Glands

For this direction of study, we found 54 articles. Fifty-one of these refer to the development of the thymus and the other three to that of the parathyroid glands (Table 2).

The 27-item checklist and the four-phase flow diagram have become widely recognized as essential tools for ensuring academic rigor in the publication of review articles. (Table 3). In this study, we utilized the flow diagram to enhance the clarity and accessibility of our findings, facilitating both interpretation and comprehension of the manuscript [25].

Most of these studies were conducted on mice and even transgenics. The second most frequent category is that of studies conducted on human cell cultures and one article presents research that took place on Zebrafish and Medaka [26].

**Figure 1:**
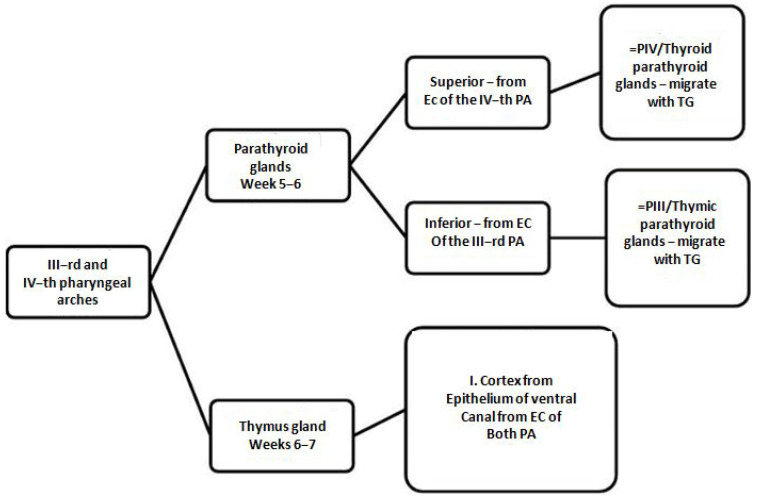
The thymus and parathyroid domains are separated from each other by less well-understood mechanisms, but involvement of neural crest cells prompts thymus migration, dragging along the inferior parathyroid glands. This representation is based on currently available experimental studies, although certain anatomical pathways remain hypothetical.

This study shows that both the thymus and parathyroid glands originate from the endoderm of the third and fourth pharyngeal pouches. The initial stages of parathyroid organogenesis are intricately associated with the development of the thymus. These organs derive from distinct regions of the same pharyngeal pouches (IIIrd and IVth) undergo a sequence of morphogenetic processes during embryogenesis to develop into separate structures [6]. While the mechanisms underlying the separation of the thymus and parathyroid domains are not fully elucidated, the involvement of neural crest cells plays a crucial role. These cells facilitate the migration of the thymus, which subsequently leads to the relocation of the inferior parathyroid glands (Figure 1).

### 3.2. Functional Relationship Molecular Research on Thymic and Parathyroid Glands

This study found 2558 (891 + 1 + 1666) articles in the order of the above-mentioned keywords (Table 4).

The articles studied in this research direction can be classified into several distinct themes:

- The role of the thymus in the immune system:
  ⮚ oncologic immunology;
  ⮚ systemic viral infections;
  ⮚ organ transplantation;
  ⮚ maternal–fetal tolerance.
- The role of the thymus in maintaining the homeostasis of lipid and carbohydrate metabolism.
- The evaluation of the possibilities of regaining thymic functions.
- The role of the thymus in wound repair.
- The role of Ca^+^ channels in thymic function.

Calcium ions (Ca^2+^) are a vital second messenger in T lymphocytes that regulate a wide range of important events, such as maturation, homeostasis, activation, and apoptosis. It enters cells through CRAC, TRP, and CaV channels, and mutations in these channels lead to T-cell dysfunction, including upregulation of several inhibitory receptors, hallmarks of T-cell exhaustion. This demonstrates the physiological importance of CaV in maintaining a resilient immune system. It acts on immunotherapy agents that enhance K^+^ channel activity, (Ca^2+^) fluxes, and CD^8+^ T-cell chemotaxis in HNSCC patients, with a unique pattern of response in responders that leads to enhanced cytotoxic T-cell functionality [27,28].

All of the data mentioned above are summarized in Table 5.

**Figure 2:**
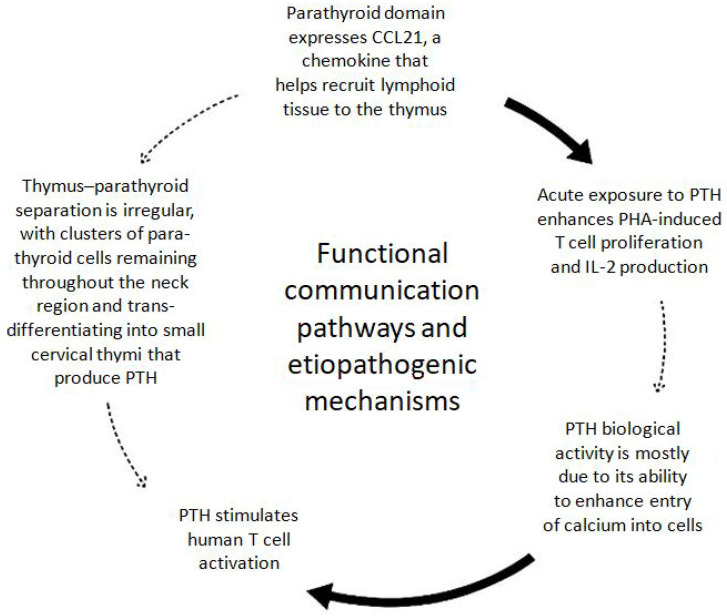
Functional communication pathways between parathyroid and thymus glands; dashed lines indicate proposed or hypothetical interactions not yet fully supported by experimental evidence. The illustrated mechanism is proposed as a conceptual model and has not yet been fully validated in vivo.

All the results on this topic are illustrated in Figure 2.

### 3.3. Etiopathogenic Relationship Molecular Research on Thymic and Parathyroid Glands

For the retrospective study on this topic, 492 articles were found (5 + 2 + 485) (Table 6).

Both articles found that common dysfunctions of the thymus and parathyroid glands lead us to congenital pathologies in which T lymphocyte dysfunction occurs due to thymic aplasia and parathyroid hypoplasia [29,30,31].

The reviewed articles have highlighted associations between thymic and parathyroid disorders, such as primary hyperparathyroidism, coinciding with thymic pathology or myasthenia gravis.

**Figure 3:**
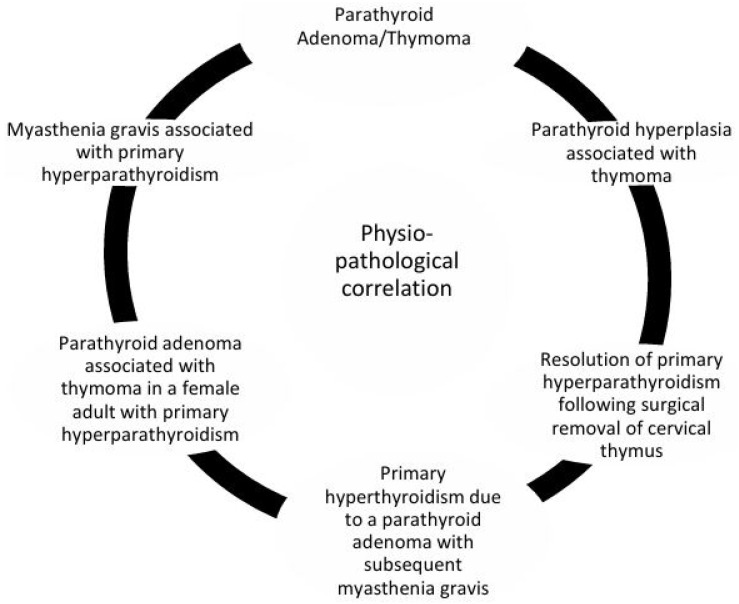
Physio–pathological correlation between parathyroid and thymus glands. This representation is based on currently available experimental studies, although certain anatomical pathways remain hypothetical.

These associations suggest a bidirectional influence in which thymic pathology could disrupt parathyroid hormone production (Figure 3).

It can, thus, be stated that the thymus could influence parathyroid function through embryological connections, shared signaling mechanisms, residual parathyroid cells in thymic tissue, and immune-mediated pathways. Understanding these interactions may help uncover links between thymic diseases, calcium homeostasis, and conditions such as myasthenia gravis (Table 7).

To provide a structured overview of the current evidence supporting the thymus–parathyroid connection, we compiled a summary of representative studies across developmental, functional, and pathological domains. The most relevant studies are presented in Table 8 below, highlighting their models, experimental design, and relevance to this evolving field.

## 4. Discussions

To advance a more comparative perspective, we contrasted the functional attributes and regulatory frameworks of the thymus and parathyroid glands. Despite their divergent physiological roles—immunological versus endocrine—their shared embryologic lineage suggests conserved developmental checkpoints, such as *TBX1*-dependent migration and differentiation. Structurally, both organs rely on stromal scaffolding that supports highly specialized cell populations, whether thymocytes or chief cells. Functionally, emerging evidence indicates potential intersections, as parathyroid-derived PTH may modulate thymic microenvironmental cues, while thymic mechanisms of central tolerance (via AIRE) may indirectly shape peripheral endocrine immunity. This duality positions the thymus–parathyroid relationship not merely as a developmental coincidence but as a potentially functional axis warranting further exploration.

### 4.1. Convergent Molecular Pathways in and Parathyroid Glands Organogenesis

Recent investigations into thymic organogenesis have primarily concentrated on thymic epithelial cells (TECs) and T lymphocytes. Extensive efforts have aimed at recreating thymic tissue that is both endocrinologically and immunologically functional. Among the most active research directions are single-cell approaches designed to generate thymic progenitor lines, enabling the study of their morphological and immunological maturation [42,43].

Parallel to this, molecular studies increasingly examine the mechanisms that regulate thymic functionality, particularly through “crosstalk” pathways within the glandular microenvironment. Many recent reviews, especially from the past five years, highlight the integration of genetic methodologies in studying thymic growth and activity. Central among these are the genes and ligands implicated in early thymic proliferation, which remain a significant focus in developmental immunology literature [44].

Gene networks, such as *PAX8* and *DLX,* have drawn attention for their relevance in oncologic contexts involving the thymus. Additionally, factors such as Lin28, HOXA3, and the ligand RANKL have been explored for their role in endothelial and epithelial differentiation, critical processes in TEC development. Of particular interest is the FOX gene family, which appears to serve as a molecular link between the thymus and parathyroid glands—both derivatives of the third and fourth pharyngeal pouches. This dual involvement underlines shared embryological mechanisms and opens avenues for comparative therapeutic strategies [34,45,46].

In contrast, research focused specifically on parathyroid gland development is relatively limited but nonetheless revealing. Among the three core studies identified, two (a review and an experimental paper) discuss diverse methodologies for the in vivo generation of functional parathyroid tissue [47,48]. The third article emphasizes the role of the GCM2 gene, which is crucial for the proliferation and maintenance of adult parathyroid cells, reinforcing the idea that this gland’s development also hinges on a set of tightly regulated genetic factors [6,49,50].

Taken together, these data suggest that while the thymus and parathyroid glands fulfill divergent biological roles, they are built upon a partially overlapping molecular foundation. This shared developmental platform supports a more integrated, comparative approach in future studies of glandular biology and therapy design.

### 4.2. Functional Interactions and Molecular Crosstalk Between the Thymus and Parathyroid Glands

The thymus and parathyroid glands, though functionally distinct, are interlinked by their common embryological origin and partially overlapping signaling pathways. Recent studies have proposed that this developmental proximity may result in shared molecular mechanisms that persist into adulthood and potentially influence glandular function [51].

For instance, early parathyroid expression of Ccl21—a chemokine involved in lymphoid cell recruitment—may play a role in thymic colonization during embryogenesis. Likewise, neural crest-derived signaling molecules regulate the migration and differentiation of both glands before their anatomical separation, suggesting a period of critical molecular interaction [52]. In some cases, ectopic parathyroid tissue may remain embedded within the thymus, descending into the thorax. These parathyroid remnants are functional and may influence systemic calcium balance through local PTH secretion, although more recent studies have largely overlooked this phenomenon [6].

Comparatively, circadian rhythm control appears to be a shared feature of both the parathyroid and thyroid glands, being localized to hormone-secreting cells. This suggests potential chronobiological parallels that remain to be explored in the thymus [50].

Immune-mediated signaling further supports potential crosstalk. The thymus, as a central site of T-cell maturation, may indirectly influence parathyroid function through systemic immune signals. In return, PTH has been shown to modulate T-cell activation, suggesting a bidirectional relationship between endocrine and immune regulation [53].

Traditionally viewed as a vestigial organ, the thymus has emerged as a cornerstone of immunological research, with growing relevance in genetics, epigenetics, and systemic diseases. Its impact extends to infection response, vaccine efficacy, transplantation, cancer, and pregnancy tolerance [54].

The intrathymic environment regulates T-cell differentiation via hormones (e.g., thymosin, thymulin, thymopoietin) and cytokines from stromal cells and thymocytes. These peptides, much like mature T cells, enter circulation and exert effects on peripheral tissues. The thymus’s influence on cancer biology and therapeutic outcomes remains incompletely defined, though recent studies offer promising insight [55].

T-cell-centered cancer immunotherapy, built on decades of foundational thymic research, now underpins a new era in oncology. Future progress depends on a deeper understanding of thymocyte interactions with APCs and NK cells to refine therapy strategies [56].

Viral and bacterial infections can alter thymic function either directly—by targeting thymic microenvironmental cells—or indirectly, via systemic release of cytokines and glucocorticoids. Contrary to previous beliefs, the thymus actively participates in antimicrobial defense, and chronic infections here impair T-cell differentiation and systemic immunity [57,58].

Thymic Treg cells, more so than their splenic or peripherally induced counterparts, are essential for maintaining immune tolerance, including at the maternal–fetal interface. Their specialized role in sustaining antigen-specific immune suppression underscores the thymus’s broader contribution to immunological homeostasis [59].

The thymus is also emerging as a central player in the pathogenesis of type 1 diabetes mellitus (T1DM). Prior to disease onset, thymic function enhances immune tolerance via Treg generation. Once autoimmunity is established, thymic output declines, mirroring systemic immune dysregulation. Modulating thymic activity could, thus, represent a therapeutic strategy to mitigate insulin resistance and improve glycemic control [60].

Modern interventions aim to restore thymic function using anti-inflammatory agents, TEC-based regeneration, and protocols to reverse structural damage caused by trauma, infection, or cytotoxic therapies. These strategies capitalize on the thymus’s remarkable regenerative capacity, enabling the restoration of central tolerance and immune reconstitution [61,62].

In tissue repair, thymic-derived T cells—particularly Tregs—support wound healing by modulating inflammatory responses and preventing excessive damage. Thymus daenensis oil, although plant derived and unrelated to the immune organ itself, has demonstrated antimicrobial and immunomodulatory properties that could support wound management. However, this compound should not be conflated with endogenous thymic activity in scientific discourse [31,63].

### 4.3. Etiopathogenic Interactions and Molecular Insights Linking the Thymus and Parathyroid Glands

The intricate interplay between autoimmune pathologies and thymic function is exemplified by myasthenia gravis (MG), a chronic neuromuscular disorder caused by antibodies targeting acetylcholine receptors (AChRs) at the neuromuscular junction. At the molecular level, these autoantibodies bind to the extracellular domain of AChRs, leading to receptor internalization, complement-mediated lysis, and impaired acetylcholine signaling, all of which contribute to progressive muscle weakness. Importantly, recent studies have revealed the role of regulatory T cells (Tregs) and complement pathways in modulating disease severity, thereby highlighting the thymus’s critical role in immune homeostasis [64,65].

Genetic predisposition to MG is another area where thymic involvement becomes evident. Risk genes associated with autoimmune dysregulation and impaired thymic output have been identified, linking thymic pathology to systemic autoimmunity. These discoveries have led to innovative therapies targeting specific molecular mediators, such as monoclonal antibodies directed at the complement cascade or immune checkpoint inhibitors, both aimed at attenuating the aberrant immune response and improving clinical outcomes [66].

In parallel, the etiopathogenic implications of thymic dysfunction extend toward parathyroid regulation. Pathological thymic alterations—such as those observed in thymomas—or age-related involution can perturb the local microenvironment, thereby impacting ectopic or adjacent parathyroid cell clusters. This disruption has the potential to affect PTH secretion and systemic calcium balance, offering a plausible mechanistic link between thymic pathology and parathyroid endocrine dysregulation. While still insufficiently explored, these findings invite further comparative investigation into the shared etiopathogenic axes of these embryologically related glands.

Based on the integrative evidence reviewed, we propose a unifying hypothesis that supports the existence of a dynamic and bidirectional axis between the thymus and the parathyroid glands. This relationship is anchored in their common embryologic origin from the third pharyngeal pouch and is shaped by a shared set of regulatory genes, including *TBX1*, *FOXN1*, and *GCM2*, which govern both organogenesis and cellular differentiation. At the functional level, PTH has been shown to influence T-cell activation and proliferation, while intrathymic mechanisms of immune tolerance, regulated by *AIRE*, may affect peripheral endocrine responses. The presence of overlapping stromal signaling molecules and shared surface receptors suggests a paracrine or neuroendocrine mode of communication that transcends spatial separation in adult physiology. While these interactions remain incompletely defined, this proposed model outlines a coherent framework for understanding the thymus–parathyroid axis and may serve as a conceptual foundation for future investigations aiming to delineate the molecular pathways that integrate immune and endocrine function.

## 5. Conclusions and Future Directions

The developmental connection between the thymus and parathyroid glands during early organogenesis suggests the possibility of overlapping functional roles. Extraparathyroid PTH production in the thymus, which shares a common origin with the parathyroid glands during organogenesis, has been proposed to provide an auxiliary source of PTH. Earlier studies in mice show that the normal process of parathyroid organogenesis in both mice and humans leads to the generation of multiple small parathyroid clusters in addition to the main parathyroid glands, which are the likely source of physiologically relevant “thymic PTH” [6,67]. Such studies have not been repeated in the period 2019–2025.

However, the primary physiological functions of these organs are apparently distinct. The thymus is primarily responsible for T-cell production, essential for adaptive immunity, while the parathyroid glands regulate calcium homeostasis through the secretion of PTH.

During early organogenesis, prior to the separation of the thymus and parathyroid glands, the parathyroid domain expresses Ccl21, a chemokine responsible for recruiting lymphoid cells to the thymus [68].

While *Gcm2* is exclusively required for parathyroid development, its presence after parathyroidectomy initially led to the hypothesis that the thymus might serve as an alternative source of PTH. This theory was disproven. Later studies revealed that incomplete separation of the thymus and parathyroid during development results in clusters of residual parathyroid cells throughout the cervical region. These cells transdifferentiate into small cervical thymi, producing PTH.

Research has demonstrated that thymic epithelial cells expressing only AChR subunits prime T-helper cells, which subsequently generate antibodies against thymic myoid cells. These myoid cells, in turn, express fully formed, clustered AChRs, leading to immune responses against intact muscle AChRs [69].

Recent studies have shown that PTH directly influences T-cell activity. Key findings include that PTH acts on T cells to enhance PHA-induced proliferation and IL-2 production. This effect is attributed to PTH’s ability to facilitate calcium entry into cells, a critical factor for its biological activity [70].

These findings establish a third correlation: PTH-activated T cells may indirectly contribute to the formation of AChR autoantibodies, linking parathyroid and thymic functions in immune modulation.

Our research indicates the following novel correlations:

- A parathyroid adenoma/thymoma association was observed; this coexistence of parathyroid adenomas and thymomas suggests a developmental or functional link between these glands.
- Instances of MG coexisting with primary hyperparathyroidism (PHPT) imply shared immune or endocrine regulatory pathways.
- A review of parathyroid hyperplasia in patients with thymoma highlights the potential for thymic abnormalities to influence parathyroid function.
- In adult females, cases of parathyroid adenomas co-occurring with thymomas have been documented in the context of PHPT, further linking the two organs.
- Rare cases describe PHPT caused by parathyroid adenomas that later lead to the development of MG, possibly through immune dysregulation.
- The resolution of PHPT after thymus surgical excision of the cervical thymus has, in some cases, resulted in the resolution of primary hyperparathyroidism, indicating the presence of ectopic or residual parathyroid tissue within the thymus [71].

This review collectively suggests overlapping developmental, functional, or immune mechanisms between the thymus and parathyroid glands.

The observed correlations suggest that the parathyroid gland may significantly influence thymus-related pathology and the development or exacerbation of MG. This influence appears to be mediated through mechanisms involving PTH levels, which have a known role in T-cell activation and immune modulation.

Post-Thymectomy Myasthenic Crisis (PTMC) is a severe postoperative complication with a high mortality rate. Research and clinical reports suggest that unresected thymic tissue, including ectopic cervical thymic tissue, might contribute to this risk. Elevated PTH levels, potentially arising from residual thymic tissue, may indicate a higher likelihood of PTMC. Thus, it is recommended to test pre- and post-operative PTH as an indicator for potential PTMC, guiding closer monitoring and early intervention strategies [72].

Also, elevated PTH levels have been shown to stimulate T-cell activation. This “priming” effect may play a role in immune-mediated mechanisms underlying MG. If this condition persists post-thymectomy, it could heighten the risk of MG or exacerbate existing conditions over time. These insights underline the potential value of incorporating PTH measurement into the clinical management of thymectomy patients, both pre- and post-operatively. This approach may enhance outcomes by addressing short- and long-term risks associated with both thymus pathology and MG [72].

Finally, the conclusion is that there are profound correlations between the results of the three retrospective research directions undertaken. In this sense, TEC precursors, matrix metalloproteinases and Tregs play a crucial developmental, functional and clinical role, both in the case of the thymus and the parathyroid glands.

The *FOXN* gene family is the only one involved in the organogenesis of both categories of glands. Their deletions contribute to the causation of polyglandular autoimmune syndrome type I and other complex congenital malformations. All these malformations involve the synchronous manifestations of thymic and parathyroid hypofunctions, most often caused by the agenesis of these glands.

These results lead us to the hypothesis that there is a much closer functional and clinical interrelationship between the thymus and the parathyroids that could imply the existence of direct communication pathways between them. Large studies are needed, between different research centers, on cohorts of patients to prove or disprove this supposition.

Given the emerging evidence that PTH can modulate T-cell activation, it becomes increasingly relevant to explore this pathway in experimental settings. Future studies might consider co-culture models involving primary human T lymphocytes and PTH-treated epithelial or stromal cells, to assess downstream effects on cytokine production, activation markers, and T-cell polarization. In parallel, animal models with conditional knockout of the PTH receptor in lymphoid tissues could help clarify causality and shed light on the broader immunoregulatory impact of PTH. These approaches may contribute not only to a better understanding of autoimmune processes but also to identifying novel therapeutic targets at the interface between endocrine and immune systems.

## Figures and Tables

**Table 1:** Results of the databases articles on thymic organogenesis; 58 articles were identified and 7 additional records were retained after refinement filters. To avoid redundancies, overlapping results across search iterations were screened manually and duplicates were removed.

Search Query	Publication Years	Filters Applied	Date Run	Results
Parathyroid gland organogenesis	2024, 2023, 2022, 2020, 2019	Article or Review Article, All Open Access	24 January 2025, 15:23:07 GMT+0200	3
Thymic organogenesis	2024, 2023, 2022, 2021, 2020, 2019	Article or Review Article, All Open Access, English	24 January 2025, 15:13:37 GMT+0200	51
Thymic organogenesis	2024, 2023, 2022, 2021, 2020, 2019	Article or Review Article, English	24 January 2025, 15:13:25 GMT+0200	57
Thymic organogenesis	2024, 2023, 2022, 2021, 2020, 2019	Article or Review Article	24 January 2025, 15:13:19 GMT+0200	58

**Table 2:** Summary of information highlighting the number of records identified, removed, assessed, and ultimately included in the review.

Records Removed Before Screening	Duplicate Records Removed	0
Automatically removed records (ineligible)	0	
Records removed for other reasons	0	
Screening	Records subject to screening	64
Excluded records	1	
Obtaining reports	Reports requested for evaluation (after record exclusion)	63
Reports that could not be obtained	10	
Eligibility	Reports assessed for eligibility	53
Excluded reports	0	
Included	Studies included in the analysis (review)	53
Reports of the included studies	53	

**Table 3:** The main research directions derived from all these 54 articles are summarized and presented.

Criteria		Thymus	Parathyroid
Biomolecule(s)	Material and Methods	Results	
TEC	Mouse single-cell transcriptional landscape of non-hematopoietic cell	characterization of cell lineage differentiation, maturation, and temporal dynamics	
non-TEC stromal cells, TCR, ETPs, Treg, TRAs, FRCs	Review	better understanding of the cellular and molecular basis of the entire set of thymic stromal cells insights toward the in vivo reconstitution of the thymus	
TEC, ETPs, transcription factors *Foxn1* and Bcl11b, chemokine Ccl25a	Review on Zebrafish and Medaka in vivo imaging and loss-of-function analysis transcription factors *Foxn1* and Bcl11b chemokine Ccl25a	insight into the general principles of vertebrate T-cell development and thymopoiesis	
Thymic Crosstalk, Regenerative Pathways, RANKL	Review	Strategies to promote thymic function	
*PAX8*, *DLX* Genes	Review	Cancer therapy,	
Single-cell RNA-seq	droplet-based (10× Genomics Chromium System) Transcription RNA Sequencing [STRT-seq])	transcriptomic profiling of thymic organogenesis	
TEC, TEPC,	Mice single cell (sc) RNAseq lineage-tracing Tamoxifen administration Microdissection of fetal thymic lobes Cell process by Flow cytometry IHC Statistical models	fully defined medium able to support different TEC differentiation states, and of cTEPC and mTEPC-specific differentiation conditions may produce functional TEC from pluripotent stem cells (PSC) or by reprogramming	
TEC, Lin28, FOXC genes family	transgenic mice Flow cytometry analysis and antibodies Immunofluorescence BrdU incorporation and Annexin V staining RT−PCR and Q-PCR Sex steroid ablation Statistical analysis	Lin28 is regulating the development and differentiation of TECs by modulating MHCII expression and TEC proliferation throughout thymic ontogeny and involution	
NOTCH signaling, *Foxn1Cre*; Rbpjfl/fl and Foxa2Cre; dnMAML	Mice FTOC in the presence of a NOTCH inhibitor	NOTCH as a potent regulator of TEPC and mTEC fate during fetal thymus development	
Endoderm of 3PP transcription factor *HOXA3*, hESCs	Human stem cells mimicking developmental queues with Activin A, WNT3A, retinoic acid and BMP4 qRT-PCR Immunofluorescence assays Flow cytometry analysis Western blotting analysis Knockdown assay by siRNA Cell proliferation cell cycle and apoptosis analysis kit RNA-seq DESeq2 software 138.3.to identify differentially expressed genes GSEA ChIP Assay Kit Statistical analysis	*HOXA3* functioned as the on-off switch to regulate the development of hESC-derived 3PP endoderm	
NF-κB, Tregs	Mice Ex-vivo cell isolation Flow cytometry Bi-parental bone marrow chimera generation Ex-vivo cell sorting Treg suppression In vitro iTreg generation In vivo parking experiment RNA-seq analysis Statistical analyses	p100 normally restrains RelB-mediated Treg activation, and in the absence of p100, p50-RelB dimers can contribute to Treg activation	
In vivo generating PTGs from the PSCs of the patient Genes Related to PTG Development In vivo generation of PTGs from rodent PSCs	Review		current achievements and challenges in present and future PTG regenerative medicine
Single-step blastocyst complementation	mESCs CRISPR-Cas9-mediated zygote genome editing BC		BC can produce functional endocrine organs and constitute a concept in treatment of hypoparathyroidism
*GCM2*	Knockout mouse assay using Tamoxifen solution Genotyping by genomic DNA PCR SPOTCHEM D-02 for total Ca and P analysis PTH measured by Mouse PTH 1–84 ELISA Kit Paraffin section in situ hybridization Rabbit Ki-67 and PCNA Apoptotic signals detected by TUNEL assay Statistical analyses		*Gcm2* plays a prominent role in adult parathyroid cell proliferation and maintenance

**Table 4:** Summary of information highlighting the number of records identified, removed, assessed, and ultimately included from functional relationship molecular research on the thymic and parathyroid glands topic.

Phase	Description	Number (No.)
Identification	Records identified from databases	3703
Records identified from registers	0	
Total records identified	3703	
Removed before screening	Duplicate records removed	0
Records removed by automation tools	0	
Records removed for other reasons	0	
Screening	Records screened	3703
Records excluded	4048	
Reports sought for retrieval	(Not explicitly shown, inferred total)	2664
Reports not retrieved	57	
Eligibility	Reports assessed for eligibility	2607
Reports excluded: not in English	42	
Reports excluded: no open access	9	
Total reports excluded	51	
Included	Studies included in review	2558
Reports of included studies	2558	

**Table 5:** Main results of the retrospective study regarding functional relationship molecular research on thymic and parathyroid glands. All the articles are summarized by the main topic and presented in the table below.

Criteria		Thymic Function	Parathyroid Function
Biomolecule(s)	Material and Methods		Results
prothymosin alpha, thymosin alpha 1, thymosin beta 4 and thymosin beta 10, thymulin and thymopoietin	Review	activate the immune system through several mechanisms and signaling pathways, including stimulation of T-cell differentiation and maturation, activation NK and DC cells, and induction of the release of proinflammatory cytokines	
TF5	mass spectrometry	Increase T-cell numbers and functions	
Tα1	Review	Role as immune-enhancing, immune-modulating, and immune-restoring agent Activation of TLR-2 and 9 Stimulation of IL-2,-10, 12, IFN-α and -γ Activation of DC, NK cells and macrophages Inhibitation of viral replication Protection agains oxidative stress Inhibition of IL-1β and TNF-α	
Tβ4	Review	Actin polymerization Cell migration and crosstalk signaling Collagen deposition Tissue repair Supressing expression of TNF-α Ferroptosis modulation	
TP	Review	TEC differentiation	
TH	Review	TEC differentiation Anti-proinflammatory Activation of NK cells Thymus–Pituitary axis transmitter between the neuroendocrine system and the immune system increase the production of GH, PRL, TSH	
TFX^®^	Review	inhibition of herpes simplex virus type 1 replication inhibition of proinflammatory cytokine production by LPS-stimulated macrophages	
Treg, tTreg, pTreg, iTreg	Transgenic mice Induction of pTreg cells in vivo Treg cell depletion and adoptive transfer of Treg cells Isolation of thymus, spleen or LN mononuclear cells by mashing through a 40 μm cell strainer Centrifugation of blood samples on a Ficoll-Hypaque Premium Filtering decidua mononuclear cells Magnetically depleted Non-CD4+ T Treg cells were sorted by a FACSAria cell sorter Flow cytometry RT-PCR measuring of mRNA levels of chemokines Western blot Chemotaxis experiments Statistical analysis	In vitro generated iTreg cells may also play a role in maternal-fetal tolerance	
intestinal microbiota	Review	molecules and metabolites derived from the intestinal microbiota impact T-cell ontogeny	
RANKL, TNFR	Review	RANKL signaling via RANK is the master factor for osteoclastogenesis mTECs act as mediators of the central tolerance process by which self-reactive T cells are eliminated while regulatory T cells are generated	
S1P, S1PR1	in vitro primary human thymocytes and in vivo and ex vivo humanized mice Thymocite line culture Mice renal implantation of human fetal thymus/liver Flow Cytometry and S1P Exposure RT-qRT-PCR Statistical Analysis	Circulating thymocytes that are not functionally mature from the thymus to peripheral blood and lymphoid organs may have implications for the immune function of PLWH	
NOTCH 1 signaling	Medaka In vivo reverse genetic approaches and whole-thymus live imaging	Notch1 controls the migratory behavior of thymocytes through controlling the chemokine receptor Ccr9b and thereby influence the T-cell receptor (TCR) activation	
CD31-positive naive T-cell differentiation status of the memory of TEC	Human PBMC isolation Expression on either CD4 or CD8, CCR7 and the absence of CD45RO	severe reduction in circulating naïve T cells because of a decrease in recent thymic emigrants is highly associated with all-cause mortality after renal transplantation	
Interleukin-22 BMP4, BMP4R antagonist Noggin KGF/FGF-7 RANKL	Review	Molecular mechanisms for endogenous thymic regeneration Strategies of Thymic Regeneration by Cell Therapies and Bioengineering, Modulation of Hormones and Metabolism, using Thymic T Cell Precursors and Bone Marrow Progenitors, Targeting Non-Hematopoeitic Cells	
inflammatory-immune cells inflammatory cytokines insulin resistance Th1 master transcription factor Th17 cells *AIRE* insulin-related peptides in the thymus Ins1 and Ins2 genes NADPH oxidase IL-10, TNF-α CCL2, *FoxN1*,	Review	changes of TEC could induce autoimmune diabetes T1DM is pa complex TEC autoimmune disease in which the pancreatic insulin-producing β-cells are selectively destroyed by the immune system thymus function have potential impact on insulin resistance T2DM is induced by chronic inflamatory diseases and high cytokine levels	
adipokines leptin and ghrelin sj-TREC ApoB	Review	Thymus has a significant role in the development of atherosclerosis and metabolic health	
C57BL/6J UCP1 *TBX1*	Stromal Cell Isolation DC Isolation Measurement of Mitochondrial H2O2 In Vitro Stimulation of Splenic T CellsImmunofluorescence Microscopy from Cytospin Autophagy Analysis by Flow Cytometry T Cell Clonal Deletion Chemokine Expression qRT-PCR Reciprocal BMT Antinuclear Antibody	Thymus role in diminished expression of tissue restricted antigens in thymus (ApoB antigen), decreased frequency of cells undergoing clonal deletion in thymus, appearance of ApoB-specific T lymphocytes in circulation Tregs Lymphocytes Control Atherogenesis	
CD4(+)CD25(+)CD127(low) CD45RO(−) CD45RA(+)CD31(+) TNF concentration IL-10/TNF ratio IL-2, IL-6, IL-8, IFN-γ, TNF-α and IL-1β CD4+Foxp3+Helios+ Tregs HDL-C	ApoE−/− mice depleted of Tregs LDLR−/− mice depeleted of Tregs CAD patients	Thymus generates atheroprotective natural T regulatory cells in wound healing	
thymic microenvironment	Review	TECs migrate to the and exert specialized effector functions and orchestrate the immune responses against tumor cells, pathogens and self-antigens Tα1 accelerates the replenishment and maturation of macrophages; block the intratumoral accumulation of myeloid suppressor cells; restrain tumor growth by its proapoptotic and anti-proliferative properties Tβ4 decreases proliferative and migratory capacities of tumor cells Tβ10 inhibites tumor cell invasion and metastasis type I NKT cells promote anti-tumor immunity and type II NKT cells suppress it	
Ca^2+^ fluxing abilities KCa3.1 and Kv1.3	Isolation of PBMCs Tumor single-cell suspension and TIL isolation Electrophysiology Ca^2+^ measured by a TCR-independent/ion channel-dependent method Flow cytometry Ion channel antibody specificity Statistical analysis	Cancer therapy pembrolizumab elicits an ion channel and functional phenotype in responder patients’ cytotoxic T cells that is conducive to a heightened ability of these cells to chemotax and kill cancer cells	
VDCCs, pore-forming CaV (α1)-, the β regulatory-	infection mice models Splenocyte Isolation Flow Cytometry Surface Staining Ca^2+^ Flux Assay Statistical Tests	diminished Ca^2+^ flux suggests the exhaustion of T lymphocytes in CaV1.4-deficient mice, provided by assessing T lymphocyte functions, such as cytotoxicity L-type Ca^2+^ channel deficiencies often lead to a phenotype that includes impaired TCR signaling, resulting in diminished T cell effector functions and reduced T-cell survival	
gene *AIRE* (21q)	Observational study on type 1 autoimmune polyendocrinopathies of the patients		If transcription of autoantibodies are inhibited, specially at the thymus level, parathyroid agenesis appears

**Table 6:** Summary of information highlighting the number of records identified, removed, assessed, and ultimately included from etiopathogenic relationship molecular research on the topic of thymic and parathyroid glands.

Stage	Description	Number (No.)
Identification	Records identified from databases	2805
Records identified from registers	0	
Total records identified	2805	
Exclusions before screening	Duplicate records removed	0
Records removed by automated tools	0	
Records removed for other reasons	0	
Screening	Records subject to screening	2805
Records excluded	328	
Obtaining reports	Reports requested for evaluation	715
Reports that could not be obtained	20	
Eligibility	Reports assessed for eligibility	502
Reports excluded (not in English)	7	
Reports excluded (no open access)	3	
Total reports excluded	10	
Included	Studies included in the review	492
Reports of included studies	492	

**Table 7:** All the articles are summarized by the topic and this table presents the results of the retrospective study related to etiopathogenic relationship molecular research on thymic and parathyroid glands.

Criteria		Thymic Function	Parathyroid Function
Biomolecule(s)	Material and Methods	Results	
22q11.2 deletion	*TBX1* qPCR probe qPCR TaqMan technique MLPA R Studio 2024.12.1+563 for statistical analysis	The use of the same sample for qPCR and MLPA is a possible newborn screening method for 22q11.2 deletion syndrome in CCHD patients	parathyroid and thyroid gland hormonal dysfunctions
22q11.2 deletion	Review	genetic modifiers and environmental factors, as well as the impact of hemizygosity on the remaining allele, contribute to the intricate genotype–phenotype relationships	parathyroid and thyroid gland hormonal dysfunctions
Oxidative Stress 22q11.2 deletion	Review	autoimmune disorders due to immunodeficiency and immune dysregulation caused by thymic dysfunction and hypoparathyroidism/neonatal hypocalcemia related to parathyroid abnormal development consistently contribute to the clinical phenotype	thymic hypoplasia or aplasia with consequent immune deficiency, cardiac malformations, hypoparathyroidism
CDNA, RNA,	Transgenic Mice Immunofluorescence RNA Extraction and Quantitative PCR Statistical Analysis NanoDrop	APS-1 is an inherited autosomal disorder caused by mutations in the autoimmune regulator (*AIRE*) gene, leading to adrenocortical failure, hypoparathyroidism, and chronic mucocutaneous candidiasis due to impaired central tolerance in the thymus.	HP
21q22.3 gene, *AIRE*, AC-Abs, STC-Abs, Antibodies to germline cells, Antibodies to CYP Enzymes, Epitope Targeting, Adrenal antibody,	Review	APS1 is based on the classic triad idiopathic hypoparathyroidism (HPT)—chronic mucocutaneous candidiasis—autoimmune Addison’s disease non-dependence on *AIRE* of the main adrenal self-antigens in the human thymus raises questions about the intrinsic mechanisms that trigger autoimmunity in APS1	HP
22q11.2 deletion syndrome, TBX, *FOXN1*, scRNA-Seq	Review	mesenchymal cells were causal to the small embryonic thymuses in the 22q11.2DS mouse models	Hypoplastic embryonic thymuses from 22q11.2DS mouse models maintain normal thymopoiesis. 22q11.2DS causes congenital malformations affecting the thymus, heart, and parathyroids
Nicotinic AChR, AChR antibodies IgG1 and IgG3, membrane attack complex, MuSK, Lrp4 Antibody, B Cell Tolerance, TECs, Cytokines, Tregs	Review	autoantibodies disrupt cholinergic transmission between nerve terminals and muscle fibers by causing downregulation, destruction, functional blocking of AChRs, or disrupting the clustering of AChRs in the postsynaptic membrane	

**Table 8:** Key studies that demonstrate the developmental, functional, or pathogenic interplay between the thymus and parathyroid glands. The table includes the author(s), year, study model, and experimental findings, and how each study contributes to understanding the thymus–parathyroid axis.

Author(s)	Year	Study Model	Experimental Evidence	Relevance to Thymus–Parathyroid Axis
Blackburn and Manley [32]	2004	Mouse model	Conditional deletion of *Foxn1* and Gcm2 disrupted thymus and parathyroid development	Shows shared embryologic origin and gene dependency
Gordon et al. [33]	2001	Mouse mutant (nude/SCID)	Lack of *Foxn1* impairs thymic epithelium; adjacent parathyroid displacement observed	Functional and spatial link during development
Fu et al. [34,35]	2003	Human fetal tissue	HOXA3 and *TBX1* co-expression in pharyngeal endoderm	Identifies key genes guiding organ separation
Liu et al. [6]	2010	Zebrafish model	Dual expression of *GCM* genes in 3rd pouch derivatives	Evolutionary conservation of thymus–parathyroid ontogeny
Wu et al. [36,37,38]	2001	Human adults	Persistent thymic -parathyroid remnants near thyroid	Suggests incomplete organ separation clinically
Neves et al. [8]	2012	In vitro co-culture	PTH modulates thymocyte activation in stromal microenvironments	Possible endocrine–immune interaction
Perniola et al. [39]	2018	APS-1 patient data	AIRE mutation disrupts thymic tolerance; leads to hypoparathyroidism	Pathogenic link via shared central tolerance
Gao et al. [31,40,41]	2013, 2024, 2023	Mouse knockout (*Tbx1*−/−)	Thymus and parathyroid aplasia with cardiac outflow defects	Reinforces *TBX1* as shared morphogenetic factor

## Data Availability

Data are contained within the article.

## Supplementary Materials

The following supporting information can be downloaded at: https://www.mdpi.com/article/10.3390/ijms26136000/s1.

## Abbreviation

TEC	thymic epithelial cell
non-TEC stromal cells	Non-Epithelial Thymic Stromal Cells
TCR	T cell receptor
ETPs	Early T-cell progenitors
Treg	regulatory T cells
TRAs	peripheral tissue-restricted antigens
FRCs	fibroblastic reticular cells
ETPs	early T-cell progenitors
RANKL	Receptor activator of nuclear factor kappa B ligand
*PAX8*	Paired-Box Gene 8
TEPC	thymic epithelial progenitor cell
IHC	Immunohistochemistry
FTOC	fetal thymic organ culture
3PP	third pharyngeal pouch
hESCs	human embryonic stem cells
GSEA	Gene set enrichment analysis
ChIP Assay Kit	Chromatin Immunoprecipitation (ChIP) Assay Kit
NF-κB	nuclear factor-kappaB
Tregs	Regulatory CD4 T cells
PTGs	parathyroid glands
PSCs	pluripotent stem cells
mESCs	mouse embryonic stem cells
BC	blastocyst complementation
*GCM2*	Glial cells missing homolog 2
TF5	Thymosin Fraction V
Tα1	Thymosin alpha 1
Tβ4	Thymosin beta 4
TP	Thymopoietin
TH	Thymulin
TFX^®^	Thymus factor X/Thymostimulinum
tTreg	thymus-derived Treg
pTreg	peripherally derived Treg
iTreg	in vitro induced Treg
TNFR	novel tumor necrosis factor receptor
S1P	HIV-1 on Sphingosine-1-phosphate
S1PR1	S1P receptor 1
PLWH	people living with HIV
RT-qRT-PCR	Real-Time Quantitative Reverse-Transcription PCR
PBMC	peripheral blood mononuclear cells
BMP4	Bone Morphogenic Protein 4
KGF/FGF-7	Keratinocyte growth factor
*AIRE*	Autoimmune regulatory protein
CCL2	C-C Motif Chemokine Ligand 2
*FoxN1*	Thymus transcription factors forkhead box N1
T1DM/T2DM	Type 1 and 2 diabetes mellitus
adipokines	cytokines produced by adipose tissue
sj-TREC	T cell receptor excision circles
ApoB	apolipoprotein A
UCP1	mitochondrial uncoupling protein
*TBX1*	T-box transcription factor
NKT	Natural killer T cells
KCa3.1 and Kv1.3	potassium (K^+^) channel
PBMCs	Peripheral blood mononuclear cells
VDCCs	voltage-dependent Ca^2+^ channels
MLPA	multiplex ligation-dependent probe amplification
CCHD	Conotruncal Congenital Heart Disease
APS-1	Autoimmune polyglandular syndrome type 1
HP	hypoparathyroidism
AC-Abs	Antibodies to adrenal cortex
STC-Abs	antibodies to steroid-producing cells
